# Calcific Deposits in Tooth Pulps

**Published:** 1886-06

**Authors:** Edgar D. Swain


### Independent Practitioner.

VOL. VII. JUNE, 1886. NO. 6.

### Original Communications.

NOTE.—No paper published or to be published in another journal will be accepted for this department. All papers must be in the hands of the Editor before the first day of the month preceding that in which they are expected to appear. Extra copies will be furnished to each contributor of an accepted original article, and reprints, in pamphlet form, may be had at the cost of the paper, press-work and binding, if ordered when the manuscript is forwarded. The Editor and Publishers are not responsible for the opinions expressed by contributors. The journal is issued promptly, on the first day of each month.

### CALCIFIC DEPOSITS IN TOOTH PULPS.

BY EDGAR D. SWAIN, D. D. S.

READ BEFORE THE ODONTOLOGICAL SOCIETY OF CHICAGO.

The pathological changes to which the tooth pulp is liable are many, and their importance commensurate to that of the organ itself. These changes vary in condition from those which are in the highest degree morbid and reparative, to those which entail the total destruction of the organ, with a corresponding injury to the nutrition and vitality of the tissue surrounding and most intimately connected with it, the dentine.

The changes which I shall discuss this evening come more directly under the head of calcifications, covering such as are better known as osteo-dentine, reparative dentine, and calcification islands or pulp stones; the last named are supposed to be the cause of prolonged and sometimes acute odontalgia or neuralgia, but just how to account for their presence, or how to diagnose their existence in a tooth pulp not sufficiently destroyed with caries to expose the affected member, is as yet an unknown quantity among the many other accomplishments of our profession.

This is a subject which has interested me and occupied my mind more or less for several years, and I was gratified, during a recent visit to Peoria, to find that Prof. Black had commenced investigations of this subject which will, in time, give us much that will be new in this direction. I was also pleased to learn that, in a general way, my ideas on the subject were almost identical with those of that most careful observer.

During the year of 1879, when in the office of Dr. Cushing as assistant, with considerable time to dispose of, I procured from the Colton Extracting Company, as well as from other sources, three hundred and eighty teeth in various stages of disease, the majority of them being mere wrecks of once beautiful structures.

I now regret that my observations in this direction were not more carefully made, and that I did not study more thoroughly all the conditions apparent in these past-members of the oral cavity. But at that time I had no idea how valuable microscopic observations of so many diseased teeth would be to me, and, in fact, I had not the knowledge necessary to bring them to a successful termination. But to return to my specimens. I divided them into four different lots, my only guide in classifying them being the external evidences of disease. Two hundred were badly decayed, with pulps either largely exposed, or such as would have been had the carious matter been removed; nearly all had evidently been the cause of terrible suffering to their former owners. I selected another hundred, less diseased, pulps not exposed, except in a very few instances where the caries was penetrating, with less superficial territory involved. The third lot consisted of sixty teeth, slightly carious, or showing worn or crushed proximal surfaces, evidences of exposed cementum to a greater or less degree, by absorption of surrounding tissue.

The fourth lot consisted of the remaining teeth, twenty in number, exhibiting few, if any, signs of disease, and had presumably been extracted in the preparation of mouth for dentures, or for the purpose of regulating remaining teeth. Understand, I was not looking for dentine of repair, transparent zones beneath the carious cavity, or for osteo-dentine, but simply for what we then called calcified pulps, or what are known as pulp stones. I proceeded then to break these teeth into pieces with a hammer and anvil, as carefully as possible, in order to preserve all fragments. You will see that the method was primitive and bungling, and not likely to result in any great amount of scientific information. The result, however, was as follows: The first two hundred produced eighteen in which I found calcific deposits of greater or less size; the third hundred furnished me with eleven more fair-sized jewels; sixty, composing the third lot, added five more of the precious gems to my collection; and lot number four gave me an addition of three. Believing that some, less affected than the thirty-seven mentioned, had escaped my observation, I concluded it fair to estimate the number thus diseased at twelve per cent., which I now believe to have been a very low estimate. About this time, if I remember rightly, Dr. Cushing procured a microscope and kindly allowed me the use of it.

I concluded to search for evidences of reparative dentine, and having read that elephants were often wounded in the tusks, and that nature healed the wound, I visited several ivory turners in search of information, and to find, if possible, a tusk that had been so wounded. I succeeded, in a measure. Mr. Thomas, then and until recently a dealer in ivory, had a specimen which had, to all appearances, been wounded and repaired. While examining this tusk we discovered within its cavity portions of the pulp desiccated, and upon tearing it away I was delighted to find a calcific deposit within, about the size of a marrowfat pea, which Mr. Thomas presented to me, together with chips from the healed tusk. Slides prepared from this nodule presented, under the microscope, the same characteristics as those from human teeth.

Mr. Salter, under the head of pulp stones, states that he secured from a dissecting room thirty-four teeth, none of which were affected with caries, but all bore evidences of some disease or injury, either past or present.

These teeth he examined carefully, securing vertical sections from most of them. He discovered that every tooth showed signs of calcific deposits in its pulp. His observations certainly show how crude and imperfect were the experiments made by myself.

In my labors with the microscope I reduced a goodly number of the pulp stones to sections, and was at first impressed by the close resemblance to normal dentine, but further examinations of most of the specimens disclosed conditions which I found were difficult to classify with normally-formed dentine. The apparent canaliculi were not those of regular formed or developed dentine, and they presented also a lamellated appearance, indicating that they were built up in layers from the center outward. Subjected to dilute hydrochloric acid, the lime was removed, but the form was retained by the matrix or organic tissue entering into the composition of the deposit, some of which, when deprived of the lime salts and thin sections cut therefrom, presented very nearly the appearance of normal pulp tissue.

Let us consider various authorities upon the causes, appearances, etc., of the formation, as well as the different theories concerning their development. Hunter speaks only of the dentine of repair in teeth worn away by attrition, and makes no mention of pulp stones.

Oudet mentions two kinds of secondary dentine, which he divides into two classes—adherent and unattached; the latter, more than likely, referring to pulp stones. Both conditions he believed to be the result of irritation to the dentine by attrition.

Heitzman, in speaking of these deposits, says: "The process leading to their formation is by no means a mere deposition of lime salts, but a transformation of the pulp tissues. That they are partly, at least, identical with lamellated dentine, and are often devoid of dentinal canaliculi."

He believes them to be the product of a low inflammation of the pulp tissues.

Wedle is of the opinion that these are formations from an inversion of the odontoblasts into the center of the pulp tissues, and explains the phenomena in these words:

"The outer surface of the pulp tissue is covered with odontoblasts, from the broad faces of which, directed outward, extend comparatively thick processes entering the dentinal canals; while the basis tissue of the pulp consists of loose connective tissue." And he maintains the theory of inversion occurring in the layers of dentinal cells; that the calcareous grains are true concrements, and occur in connection with hard, new formations, but never enter into organic union with the original dentine; they are located within the mass of the pulp tissue and are calcareous or calcifications in the connective tissue.

Upon analysis of his theory we have the anomaly of the odontoblasts joined to the dentinal canals by strong processes projecting from their outer ends, enclosed within strong walls built by themselves, becoming inverted by a force which must exist within themselves, and depositing into the connective tissue calcareous grains intended for true dentine, as would be the case if the odontoblasts maintained their normal position. Were this true we should find zones of dead or unsensitive dentine, which would be of great assistance in diagnosing the presence of these deposits within the pulp. On the contrary, teeth thus affected are often hypersensitive.

Salter says: "This change in the pulp must be looked upon as morbid in the lowest degree, being to a great extent the result of trivial causes, and never occurring unless the tooth has been, in some way, the subject of an injury or irritation; the pulp of a young tooth, perfectly matured, never exhibits this condition unless it has received some abnormal stimulation from morbid change or mechanical injury of the dentine, the most potent cause being caries." Heitzman, however, claims to have seen calcific nodules, or pulp stones, in perfectly developed bicuspids, extracted before they were fully developed.

Further, Salter conveys to the reader the idea that calcific nodules in dental pulps are almost as common as the pulps themselves. He describes pulps in which a great many appear, and the conditions which indicate the fusion of numbers of small nodules into one large one, sections from which have the appearance of numerous small lamellated bodies. He states further, that the whole of the tissues, cells, nuclei, connective tissue, blood vessels and a multitude of nerves are swallowed up by the process of calcification. I have consulted many other writers upon this subject, but consider these authorities quite sufficient for quotation. In reading a new work which Delafield & Prudden published this year (1885), I came across the following, which convinced me that the theory I am about to present was not without foundation. I will use their own language: "In calcareous degeneration there is a deposit, either in cells or in the intercellular substances, of granules composed chiefly of phosphate or carbonate of calcium; these particles, when abundant, give hardness, brittleness and a whitish appearance to the affected tissue. Sometimes large lamellated concretions are formed, usually at the seat of some old inflammatory process. Calcification occurs in parts of tissues which are dead, or in a condition of reduced vitality, the result of some antecedent morbid process, usually of an inflammatory nature." Calcific deposits, such as have been spoken of, are not confined to the tooth pulp alone. I may instance the phlebolite, a calcareous concretion found in the veins, which, according to Paget, is formed in the blood by a union of lime salts and albumen. From my observations I have arrived at the following conclusions as to their appearance, formation, and the causes which produce them :

First, pulp stones are never connected with the dentine, but there always intervenes between the two a layer resembling a dead or desiccated membrane. This membrane evidently consists of the layer of atrophied odontoblasts, and is consequently unable to extend the work for which they were intended, namely, to appropriate and deposit the lime salts which the circulatory system continues to supply. As a consequence, the salts thus liberated by osmosis are organized into islands of calcification in the body of the pulp, surrounding the fibers and vessels, thereby giving the appearance sometimes observed of dentinal canals. This class of pulp stones is not to be confounded, however, with another which so closely resembles true bone, always presenting lacunæ. These last are the result of a transformation of connective tissue cells into osteoblasts, and sometimes to the extent of entirely obliterating the pulp cavity. I have a specimen of this character, and have seen others which have led me to conclude that this kind of pulp calcification never occurs in a fully developed tooth, but commences at that period when the apicial foramen is large, and the impetus or exciting cause is derived from the osteoblasts or bone builders, which produce the cementum. Their influence over connective tissue cells transforms them into osteoblasts, which appropriate the lime salts intended for dentine and obliterate the pulp cavity by filling it with cementum.

That insoluble salts of lime are altered in their behavior by association with different organic compounds, was first discovered by Ramée, and has since been further demonstrated by Prof. Harting, of England.

It is well known that if a soluble salt of lime be slowly mixed with a solution capable of precipitating the lime, the result will be that the salts of lime will be thrown down as an amorphous powder, and sometimes in minute crystals; but if gelatine, albumen, or other similar compounds be added, the form and physical character of the precipitated lime salts are materialy changed.

M. Ramée found that if carbonate of calcium be slowly formed in a thick solution of albumen, the resultant salt assumes the form of globules, lamellated in structure. This experiment I have verified, and have been surprised at the close resemblance of these globules to a large number of pulp stones heretofore examined under the microscope.

This experiment is simple, and may be easily accomplished by any one who desires. Take a bottle of calcium water, thickened with albumen or common gum water; then breathe through a glass tube into the mass; the carbonic acid will precipitate the lime. Another method for the experiment is this: Secure a bottle of fresh carbonic acid water from any soda fountain, this to be used in lieu of the breathing. This must be added very slowly and carefully until the lime water assumes a milky appearance, which indicates the presence of carbonate of lime. If too much carbonic acid is present the bicarbonate is formed, and the experiment will prove a failure. When the mass assumes a milky appearance cork tightly and put away. In a few weeks, if the experiment be successful, there will be found at the bottom of the bottle a number of granules, and occasionally one will discover a mulberry-formed mass among them which, upon examination, appears to be a blending together of the numerous smaller globules, such as is described by Salter as sometimes found in pulp tissue.

Harting, in his observations, found that the organic material used in his experiments had also changed its character. He named the globules formed calcospherites, and learned that when subjected to the action of acid the lime salts were removed, but that they still retained their form and were then extremely resistant to the action of acids, alkalies and boiling water. He says: "For this modified albumen I propose the name of calcoglobuline, because it appears that the lime is held in some sort of chemical combination, for the least traces of lime are retained very obstinately when calcoglobuline is submitted to the action of acids. And I am satisfied," says he, "that the calcospherite has a true matrix of calcoglobuline which is capable of retaining its form and structure after the removal of the lime."

Mr. Chas. Tomes informs us that spherical forms exactly similar to those artificially produced by Harting are to be constantly seen around the thin cap of forming dentine.

Robin and Magitot describe them as occurring abundantly in the pulps of growing teeth, both human and herbivorous.

From what has been said you may readily understand the theory which I have evolved regarding the formation of the islands of calcification in the pulps of the teeth, namely; First, that the odontoblasts have become atrophied and are no longer capable of performing their normal functions.

Second, that the circulation at the time of their formation is normal and the necessary amount of lime salt is conveyed into the pulp when required either for the purpose of building dentine in tooth development, or dentine of repair, but the atrophied odontoblasts being incapable of appropriating them for either purpose, it is therefore deposited within the tissues of the pulp.

Third, that the lime salts having been passed by osmotic forces through the coats of the vessels, and not being appropriated, and no endosmotic force being present (because of the otherwise normal conditions), meeting with the necessary organic material in the form of albumen the two unite as in the experiment, and the production is a calcospherite.

It is a well established fact that fractured crystalline bodies will, under proper conditions, repair the injury; therefore, it seems to me that this fact and the experiments of Harting prove that there is something like a universal property of bodies that are naturally and orderly constructive, and that elements which enter largely into living organisms are controlled by certain existing laws which exert themselves with or without the force we call vitality; consequently, we are enabled to produce artificially the same conditions which we observe and attempt to explain upon the theory of a present vital force.

